# Lipopolysaccharide-initiated persistent rhinitis causes gliosis and synaptic loss in the olfactory bulb

**DOI:** 10.1038/s41598-017-10229-w

**Published:** 2017-09-14

**Authors:** Sanae Hasegawa-Ishii, Atsuyoshi Shimada, Fumiaki Imamura

**Affiliations:** 1 Pennsylvania State University College of Medicine, 500 University Drive, Hershey, PA 17033 USA; 2 Faculty of Health Sciences, Kyorin University, 5-4-1 Shimorenjaku, Mitaka, Tokyo, 181-8612 Japan

## Abstract

The olfactory mucosa (OM) is exposed to environmental agents and therefore vulnerable to inflammation. To examine the effects of environmental toxin-initiated OM inflammation on the olfactory bulb (OB), we induced persistent rhinitis in mice and analyzed the spatial and temporal patterns of histopathological changes in the OM and OB. Mice received unilateral intranasal administration of lipopolysaccharide (LPS) or saline three times per week, and were immunohistologically analyzed at 1, 3, 7, 14 and 21 days after the first administration. LPS administration induced an inflammatory response in the OM, including the infiltration of Ly-6G-, CD11b-, Iba-1- and CD3-positive cells, the production of interleukin-1β by CD11b- and Iba-1-positive cells, and loss of olfactory sensory neurons (OSNs). In the OB, we observed activation of microglia and astrocytes and decreased expression of tyrosine hydroxylase in periglomerular cells, vesicular glutamate transporter 1, a presynaptic protein, in mitral and tufted projection neurons, and 5T4 in granule cells. Thus, the OM inflammation exerted a detrimental effect, not only on OSNs, but also on OB neurons, which might lead to neurodegeneration.

## Introduction

It is known that the central nervous system (CNS) actively interacts with the immune system under inflammatory, as well as healthy, conditions. Using bone marrow transplantation, we and others have established that bone marrow-derived immune cells are localized to particular regions including the leptomeninges, perivascular space, and choroid plexus stroma (meningeal space)^1^, that are adjacent to the brain parenchyma, and circumventricular organs that lack the blood-brain barrier^2–5^. These regions, therefore, may represent a ‘brain-immune interface’. Since cells in the meningeal spaces respond to endotoxemia-induced systemic inflammation and release multiple cytokines that activate astrocytes at their endfeet^6–9^, the brain-immune interface may function as a relay station through which inflammatory responses are transmitted from peripheral organs to the CNS.

Olfactory sensory neurons (OSNs) are receptor cells for smell located in the olfactory epithelium (OE). Odorants are trapped by mucus covering the luminal surface of the OE and bind to odorant receptors expressed on the dendritic cilia of OSNs^10^. Unlike other sensory cells, OSNs bear axons that pass through the lamina propria and the cribriform plate and directly target the olfactory bulb (OB), the first relay station of olfactory information in the CNS^11^. In addition to odorants, the nasal cavity is constantly exposed to a wide variety of airborne environmental agents such as bacteria, viruses, molds, dusts and pollen. Due to the close proximity of the olfactory mucosa (OM; OE and lamina propria) and the OB and their direct axonal communication, this olfactory pathway has been considered a potential route of entry for environmental agents into the brain^12, 13^.

Environmental agents entering the nasal cavity can cause inflammatory responses in the OM, including the infiltration of inflammatory cells and production of inflammatory cytokines^14^. Several animal studies have reported inflammatory responses in the OB after administration of environmental toxins to the nasal cavity. For example: intranasally applied satratoxin G and roridin A, macrocyclic trichothecene mycotoxins, induce upregulation of proapoptotic genes and proinflammatory cytokines in the OB^15, 16^; smoke inhalation significantly increases the inflammatory cytokines and chemokines in the OB^17^; zinc sulfate irrigation induces degeneration of the outer and glomerular OB layers as well as marked inflammatory responses^18^; and intranasal administration of lipopolysaccharide (LPS), which is a major component of outer membrane of gram negative bacteria and induces a strong immune response, induces microglial activation in the OB^19^. Further, patients with severe persistent rhinitis have a smaller OB volume^20^. These results strongly support that the OM-OB pathway serves as another brain-immune interface that transmits the inflammatory responses from the peripheral OM to the OB. However, the effects of toxin-initiated OM inflammation on neurons in the OB have not been examined.

In the present study, we induced persistent rhinitis in mice by repeated intranasal LPS administration to determine the spatial and temporal patterns of histopathological changes in the OM and OB, and to identify cells and tissue components in the OB affected by persistent rhinitis. We report, for the first time, that LPS-initiated persistent rhinitis exerts detrimental effects on OB neurons including projection neurons. These results are indicative of inflammation-induced neuronal damage, which could lead to further neurodegeneration.

## Results

### Inflammatory cell infiltration into the olfactory mucosa

**Figure 1:**
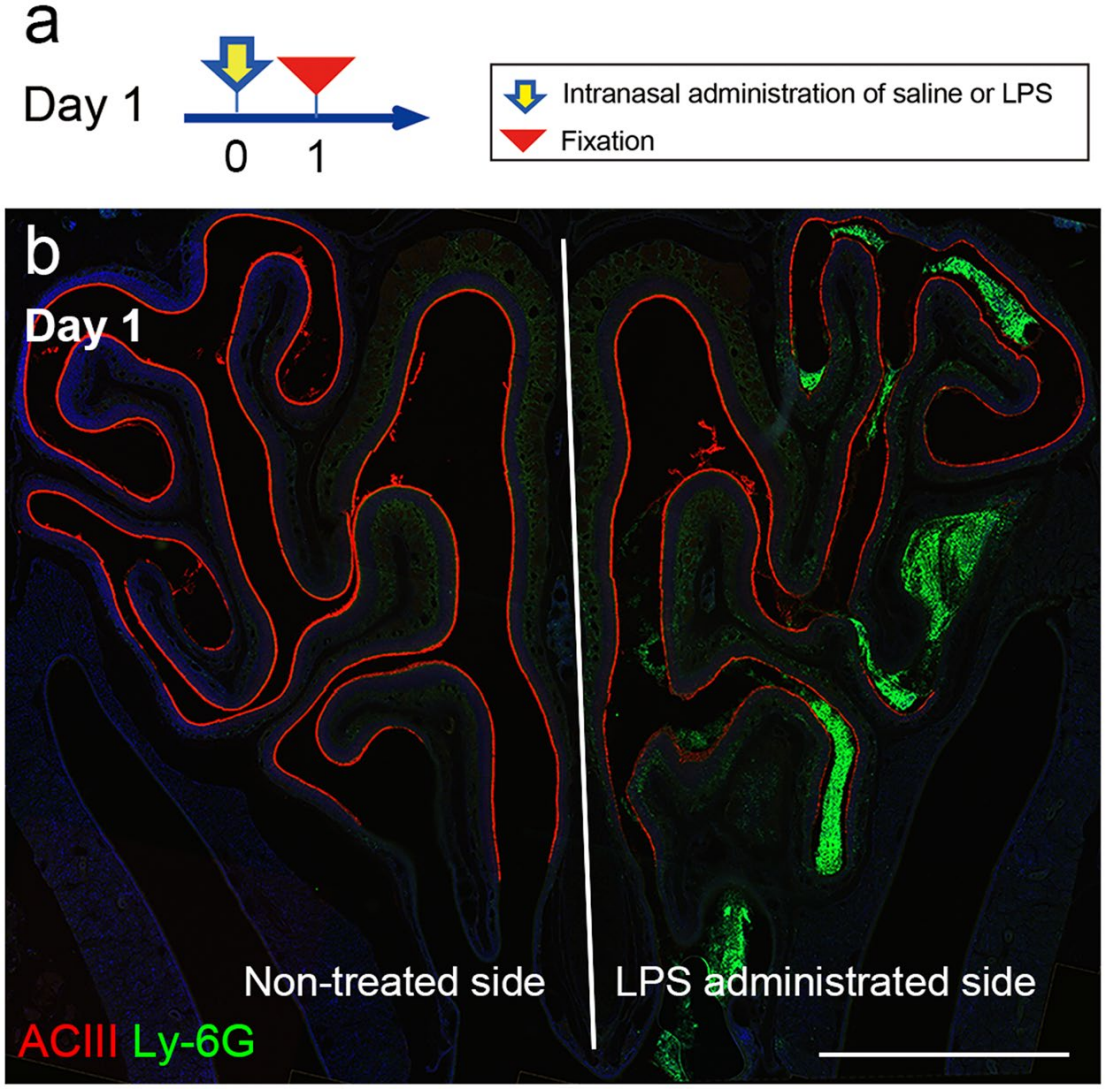
Changes in the nasal cavity 1 day after LPS administration. (**a**) Protocol for intranasal administration of LPS or saline. To cause acute rhinitis, mice received a single intranasal administration of LPS or saline and sacrificed at 1 day after the administration (Day 1). (**b**) A coronal section of the immunofluorescently stained OM shows accumulation of Ly-6G-positive cells (green) particularly in the lateral part of the LPS-treated nasal cavity. AC III immunoreactivity (red) is weaker in the lateral part of the LPS-treated OE. All nuclei are stained with DAPI (blue). Scale bar, 1 mm.

We first examined the acute effects of LPS in mice that received a single unilateral administration and were then sacrificed 1 day later (Fig. 1a). At one day following LPS administration, Ly-6G-positive cells accumulated in the OM and the nasal cavity ipsilateral to LPS administration (LPS-treated OM), while no Ly-6G-positive cells were found in the contralateral non-treated side (Fig. 1b). It was notable that Ly-6G-positive cells appeared specifically in the lateral part of the LPS-treated OE. When Trypan Blue solution was administered intranasally in order to determine the distribution of solution within the nasal cavity, the blue stain was markedly observed in the lateral part of the OE, while the medial OE showed weak staining (Supplementary Fig. 1). This indicates that easiness of solution access may be an underlying mechanism of specific immune cell infiltration to the lateral OE.

**Figure 2:**
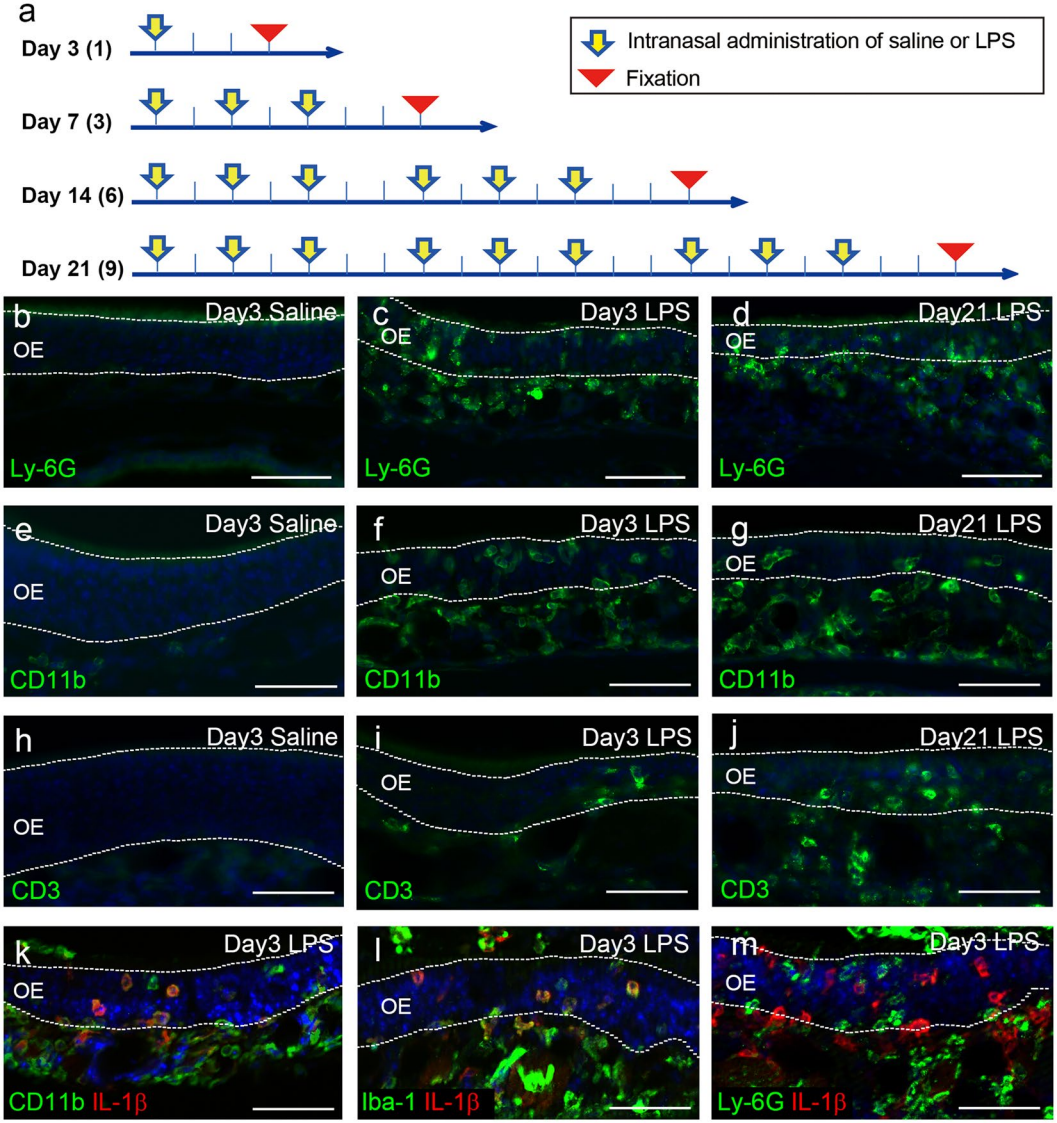
Infiltration of immune cells into the OM. (**a**) Protocol for intranasal administration of LPS or saline. To cause persistent rhinitis, mice received repeated intranasal administration of LPS or saline three times per week (every other day) and were sacrificed at 3, 7, 14 and 21 days after the first administration (Day 3, 7, 14 and 21, respectively). Total number of administration is indicated in parenthesis. (**b**–**j**) Almost no Ly-6G-positive (**b**), CD11b-positive (**e**) or CD3-positive (**h**) cells are observed in the saline-treated OM at Day 3. Many Ly-6G-positive (**c**,**d**) and CD11b-positive cells (**f**,**g**) infiltrate the lateral part of the LPS-treated OM at Day 3 and Day 21. A small number of CD3-positive cells infiltrate the lateral part of the LPS-treated OM at Day 3 (**i**) and increase in number at Day 21 (**j**). **(k**–**m)** IL-1β-positive cells (red) are observed in the LPS-treated OM; most of which are immunopositive for CD11b (k; green) and Iba-1 (l; green) but not for Ly-6G (m; green). CD11b, Iba-1 or Ly-6G positive cells (green) also extravasate into the nasal cavity. All nuclei are stained with DAPI (blue). Dotted lines indicate the boundaries of OE and mucus layer (above) or lamina propria (below). Blood vessels are observed as circular structures in the lamina propria. Scale bars, 50 μm.

Then, we examined the chronic effects of intranasal LPS administration by administering LPS to one nostril three times per week (every other day) and examined the nasal cavity 3, 7, 14 and 21 days after the first administration (Fig. 2a). To identify infiltration of inflammatory cells, the tissue was stained with antibodies for Ly-6G (a marker for neutrophils and inflammatory monocytes), CD11b (neutrophils and macrophages), Iba-1 (monocytes/macrophages) and CD3 (T cells)^21–24^. The administration of saline did not result in the infiltration of Ly-6G-positive, CD11b-positive or CD3-positive cells into the OM (saline-treated OM; Fig. 2b, e, and h). In contrast, Ly-6G-positive and CD11b-positive cells infiltrated the lateral part of the LPS-treated OM at all experimental time points examined from Day 3 to Day 21, especially around the blood vessels in the lamina propria (Fig. 2c, d, f and g), while leukocytes were rarely found in the non-treated OM. CD3-positive cells infiltrated the LPS-treated OM with increasing numbers between Day 3 and Day 21 (Fig. 2i and j); suggesting that T cells infiltrate the LPS-treated OM in a time-dependent manner. These results indicate that repeated LPS exposure was successful to induce persistent inflammation (chronic inflammation in combination with newly induced acute inflammation) in the OM.

To illuminate the macrophage and neutrophil response to LPS treatment we examined the expression of IL-1β in the OM. We found IL-1β-expressing cells in the LPS-treated OM at Day 3 (Fig. 2k–m). Double immunofluorescent staining revealed that the IL-1β-expressing cells were mostly immunopositive for CD11b and Iba-1 (Fig. 2k and l), but immunonegative for Ly-6G (Fig. 2m), indicating that they were infiltrating macrophages, not neutrophils.

### Loss of olfactory sensory neurons

**Figure 3:**
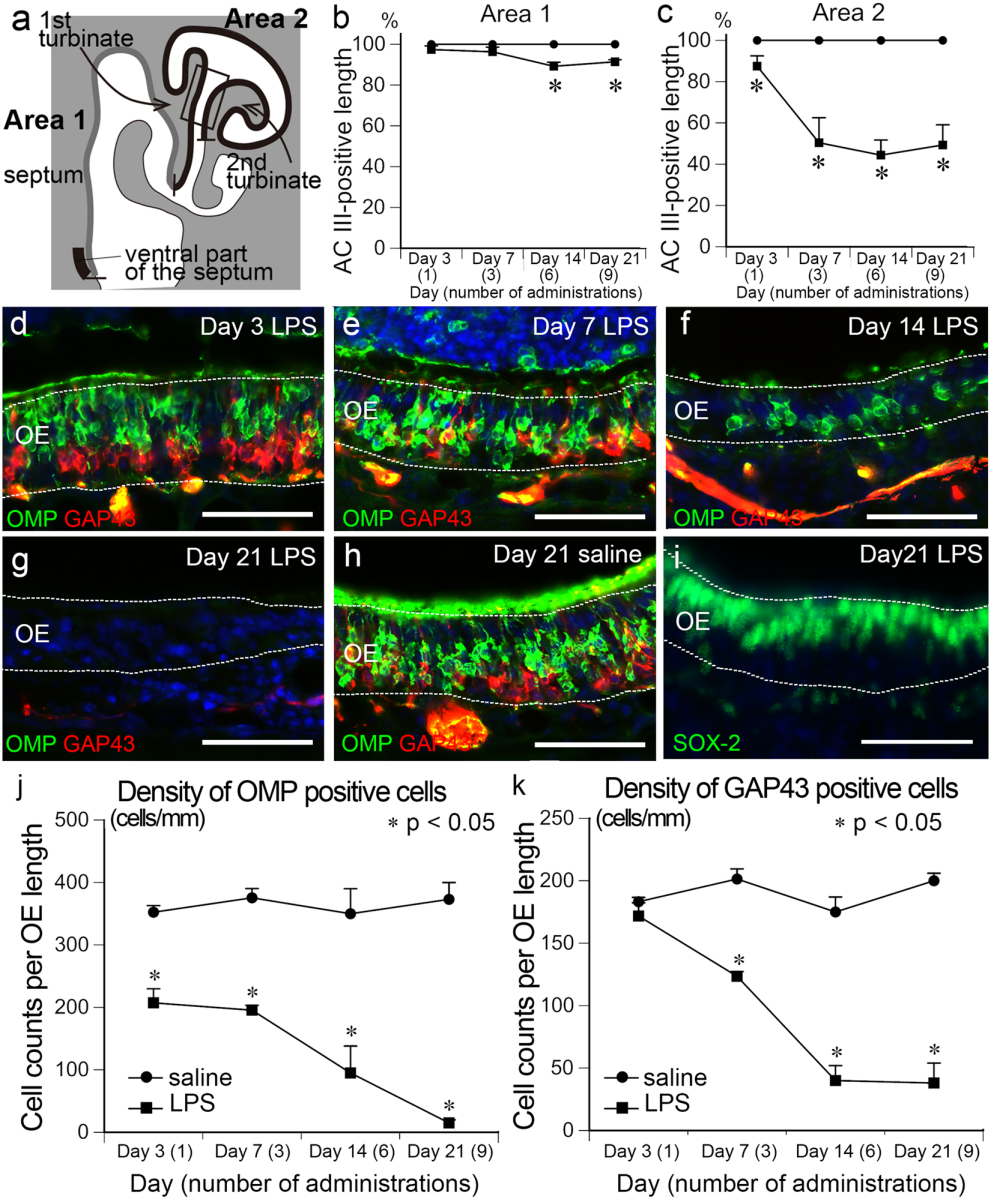
Loss of OSN in the LPS-treated OM. (**a**) The diagram represents a coronal section of the OM, including the septum, first turbinate, second turbinate and the third turbinate. Area 1 and Area 2 include the medial (gray line) and lateral (black line) portions of the OE, respectively. The thick black line indicates the most ventral part of the septum. A rectangle indicates the region that exhibited severe OSN loss and was used for quantification. (**b**,**c**) Graphs show the percentages of AC III-positive length in the Area 1 (**b**) and 2 (**c**). The AC III immunopositive area in the LPS-treated OE significantly decreased compared to saline-treated OE at Days 14 and 21 in Area 1 and at all experimental time points examined in Area 2. Statistical analyses were performed with Mann-Whitney U-test (Mean ± SEM. *p < 0.05, compared to saline-treated control at the same time points). (**d**–**h**) Both OMP-positive mature (green) and GAP43-positive immature (red) OSNs show a decline in number in the lateral part of the LPS-treated OE. (**i**) SOX-2 positive sustentacular and basal cells remain in the lateral part of the LPS-treated OE at Day 21. All nuclei are stained with DAPI (blue). Dotted lines indicate the boundaries of OE and mucus layer (above) or lamina propria (below). Scale bars, 50 μm. (**j,k**) Graphs show the densities of OMP-positive (**j**) and GAP43-positive (**k**) cells in the OE calculated from the area indicated in (**a**). The density of OMP-positive cells in the LPS-treated OE was significantly lower than that in saline-treated OE at all experimental time points examined. The density of GAP 43-positive cells in the LPS-treated OE was significantly lower than that in the saline-treated OE at Days 7, 14 and 21. Statistical analyses were performed with Mann-Whitney U-test (Mean ± SEM. *p < 0.05, compared to saline-treated control at the same time points).

We examined the effects of persistent rhinitis on OSNs by first examining a marker for dendritic cilia^25^, adenylate cyclase III (AC III). As shown in Fig. 1b, AC III was normally expressed along the luminal surface of the OM, throughout the non-treated OE. In contrast, AC III immunoreactivity was discontinuous in the LPS-treated OE, particularly in the lateral part, where Ly-6G-positive leukocytes accumulated. The region most severely damaged by LPS administration was consistently localized to OM sections cut between 360 and 720 μm rostral from the anterior tip of the OB. We quantified the AC III expression in the OE section by subdividing it into the medial part (Area 1) and lateral part (Area 2) (Fig. 3a). In the saline-treated OE, both Areas 1 and 2 had AC III-positive staining along the entire length of the OE at all experimental time points examined (Fig. 3b and c). In the LPS-treated OE, the AC III-positive length in Area 1 decreased slightly, but significantly, to 89.2 ± 2.0% and 91.4 ± 1.0% at Days 14 and 21, respectively (Fig. 3b). The decrease was primarily the result of the loss of AC III expression in the ventral part of the septum (the area indicated by thick black line in Fig. 3a). In Area 2 of the LPS-treated OE, the AC III-positive length markedly decreased to 87.5 ± 5.0, 50.4 ± 12.1%, 44.4 ± 7.3%, 49.3 ± 9.3% at Days 3, 7, 14 and 21, respectively (Fig. 3c). These results indicated that intranasal LPS administration severely impaired the OSNs in the lateral part of the OE.

Next, we examined the effect of persistent rhinitis on OSN subsets by immunofluorescent staining with olfactory marker protein (OMP), a marker for mature OSNs; and growth associated protein 43 (GAP43), a marker for immature OSNs^26^. As expected from the changes in AC III expression patterns, intranasal LPS administration produced a more pronounced loss of OSNs in Area 2 than in Area 1. In fact, almost no OMP- or GAP43-positive cells were found in the analyzed region of Area 2 at Day 21 (Fig. 3g). In contrast, SOX-2-positive sustentacular and basal cells still remained in the LPS-treated OE (Fig. 3i). We quantified the time course of OSN loss within Area 2 by counting OMP- and GAP43-positive cells at Days 3, 7, 14 and 21. In saline-treated OM, the density of OMP-positive cells (350–380 cells per mm OE length) and GAP43-positive cells (180–200 cells per mm OE length) did not change over time (Fig. 3j and k). In contrast, the density of OMP-positive cells was significantly lower in LPS-treated OE (240.1 ± 22.5 cells per mm OE length) than in saline-treated OE (351.3 ± 10.6 cells per mm OE length) as early as Day 3, and further decreased as a function of time (Fig. 3j). The density of GAP43-positive cells in LPS-treated OE did not differ significantly from those in saline-treated OE at Day 3, but decreased significantly at Days 7, 14 and 21 (Fig. 3k).

### Degeneration of olfactory sensory neuronal axons

**Figure 4:**
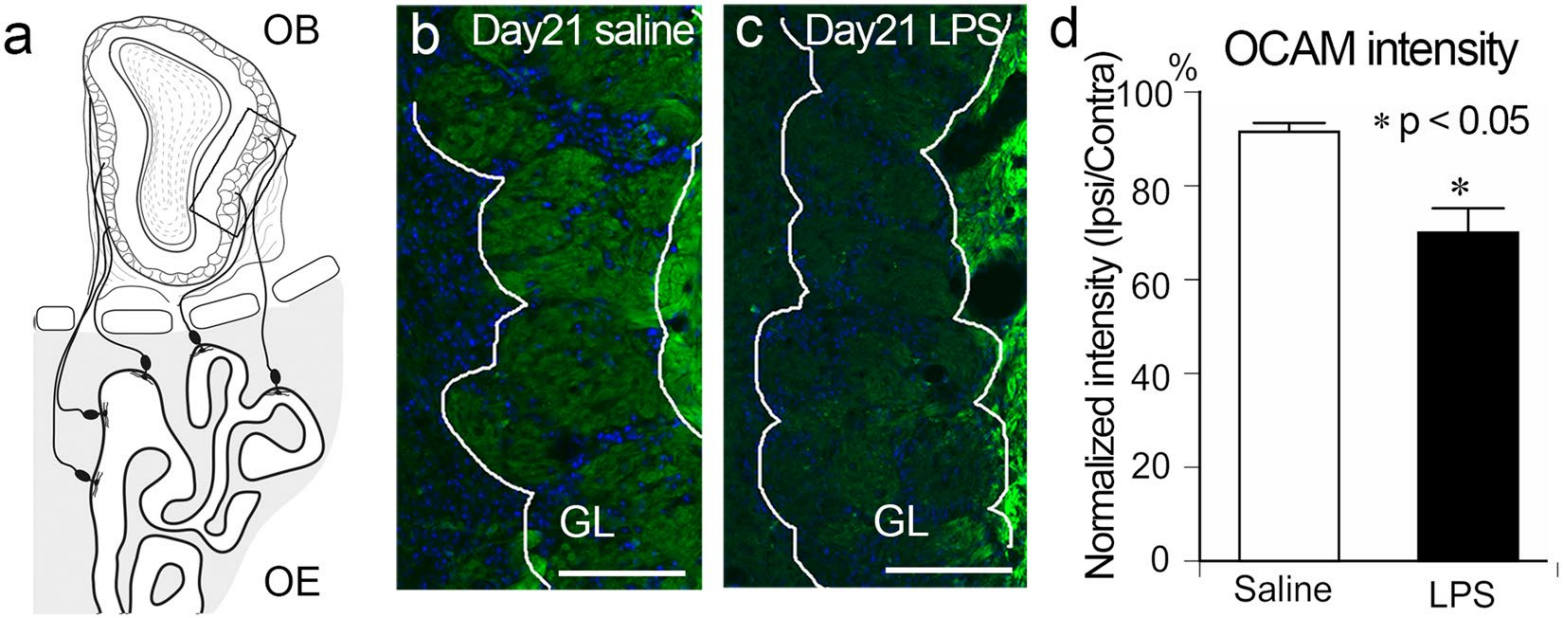
Degeneration of OSN axons in the lateral LPS-treated OB. **(a)** The diagram showing the topographical projection of OSN axons to the OB. OSNs in the lateral OE project their axons to the ventrolateral part of the OB. (**b,c**) Glomeruli in the ventrolateral OB are immunopositive for OCAM. The OCAM signal is weaker in the LPS-treated OB at Day 21 (c; green) compared to saline-treated OB (b; green). All nuclei are stained with DAPI (blue). Scale bars, 100 μm. (**d**) The normalized intensity of OCAM in the GL decreases significantly with LPS treatment compared to saline treatment. Data were analyzed with Mann-Whitney U-test (*p < 0.05, compared to saline-treated control).

The OE can be subdivided into several zones based on the expression patterns of specific molecules including odorant receptors^27^, and there is a prominent boundary between the dorsomedial region (dorsal zone) and the ventrolateral region (ventral zone). Since Area 2 was largely included in the ventral zone of the OE, we examined the effects of intranasal LPS administration on OSN axons by analyzing the expression of olfactory cell adhesion molecule (OCAM), a maker exclusively expressed by OSNs in the ventral zone^28^. OCAM immunoreactivity was seen in the olfactory nerve layer (ONL) and glomerular layer (GL) of the lateral part of the OB, the region which receives input from the OSNs in the ventral zone of the OE (Fig. 4a and b). The OCAM immunoreactivity in the GL of the LPS-treated OB was remarkably reduced compared to that of the saline-treated OB at Day 21 (Fig. 4b and c). We then normalized the OCAM intensity in the GL of the LPS-treated OB with that of the contralateral non-treated OB (see Methods). The results indicated that the OCAM expression in the GL was significantly lower in the lateral part of the LPS-treated OB (70.0 ± 5.2%) than in saline-treated OB (91.5 ± 1.9%) (Fig. 4d). This result suggests that the axons of OSNs located in the ventral zone of the OE underwent degeneration in response to intranasal LPS administration.

### Gliosis in the olfactory bulb

**Figure 5:**
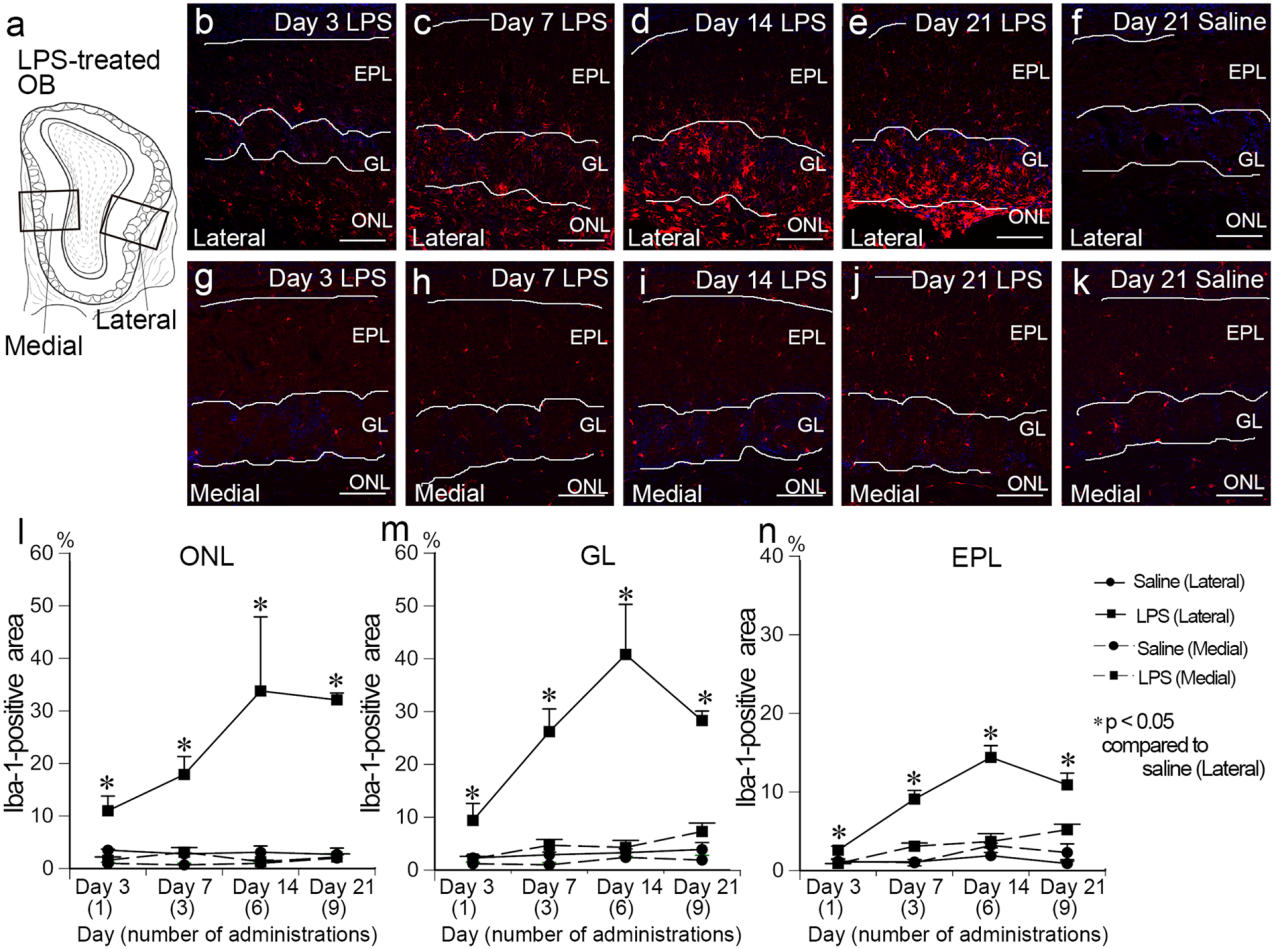
Microglial activation in the lateral LPS-treated OB. (**a**) Lateral and medial parts of the OB focused on the analysis for microglial activation are indicated by two rectangles in the diagram. (**b**–**f**) Immunofluorescent staining with anti-Iba-1 antibody (red) shows activation and accumulation of microglia in the ONL, GL and EPL of lateral part of the LPS-treated OB. (**g**–**k**) No changes in morphology and distribution of Iba-1-immunopositive microglia (red) are observed in the medial part of the LPS-treated OB. All nuclei are stained with DAPI (blue). Scale bars, 100 μm. (**l**–**n**) Graphs showing the percentages of the Iba-1-positive area in the ONL (l), GL (m) and EPL (**n**) of LPS- and saline treated OBs. Microglia are significantly activated in lateral part of the LPS-treated OB compared to that of saline-treated OB at Days 7, 14 and 21. Statistical analyses were performed with Mann-Whitney U-test (Mean ± SEM. *p < 0.05, compared to the lateral part of the saline-treated control at the same time points).

To examine the potential activation of microglia and astrocytes in the OB, we performed immunohistological studies to detect microglia and astrocytes with anti-Iba-1 and anti-GFAP antibodies, respectively. In LPS-treated OB, microglia in the lateral part exhibited larger cell bodies and thicker cytoplasmic processes (both of which reflect glial activation) than those in the medial part and were most predominant in the ONL, GL and external plexiform layer (EPL) (Fig. 5b–f). The gliosis was consistently observed in all animals in sections that were cut between 600 and 900 μm rostral from the anterior tip of the AON. The Iba-1 immunopositive area was significantly higher in the ONL, GL and EPL in the lateral part of the LPS-treated OB compared to the corresponding layers of saline-treated OB at all experimental time points examined from Day 3 to Day 21 (Fig. 5l–n). The percent of Iba-1-positive areas that were occupied by microglia in the lateral part of the LPS-treated OB reached a maximal level at Day 14 and plateaued until Day 21. The maximal percentage of Iba-1-positive areas in the lateral part of the LPS-treated OB at Day 14 were 33.8 ± 14.1%, 40.8 ± 9.5% and 14.4 ± 1.5% of the ONL, GL and EPL, respectively. These values were significantly higher than those of the saline-treated OB where less than 5% of the ONL, GL and EPL were Iba-1-positive (Fig. 5l–n). In contrast, there was no distinct change in the morphology of microglia or in the Iba-1 positive area in the medial part of the LPS-treated OB compared to the saline-treated OB (Fig. 5g–n).

Similar to findings for microglia, astrocytes became hypertrophic in the lateral part of the LPS-treated OB with greater astrocytic accumulation in the ONL, GL and EPL as a function of time (Supplementary Fig. 2). These results indicate that astrocytic gliosis progressed in the lateral part of the OB following intranasal LPS administration. There were no obvious morphological changes or accumulation of astrocytes in the medial part of the LPS-treated OB or in non-treated OB. We also observed a small number of Ly-6G-positive and CD11b-positive cells in the GL and EPL of LPS-treated OB at the lateral part at Day 1 (Supplementary Fig. 3).

### Neurodegenerative changes in the olfactory bulb

**Figure 6:**
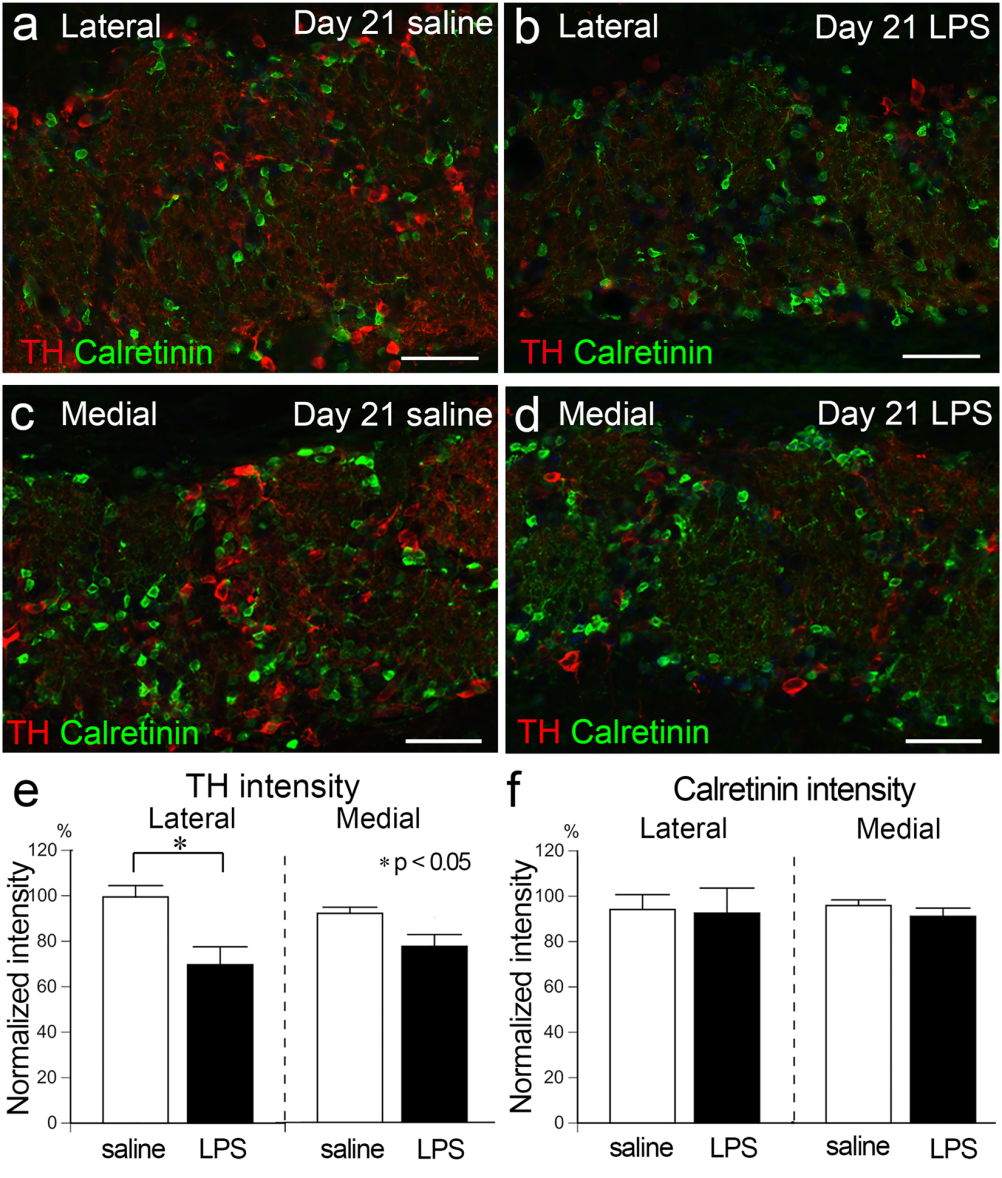
Reduction of tyrosine hydroxylase signal in periglomerular cells in the LPS-treated OB. **(a-d)** Images from the GL of saline- (**a**,**c**) and LPS-treated (**b**,**d**) OBs stained with TH (red) and calretinin (green). TH signal, but not calretinin, is weaker in the lateral part of LPS-treated OB (**b**) compared to that of saline-treated OB (**a**) at Day 21, but not in the medial part (**c**,**d**). All nuclei are stained with DAPI (blue). Scale bars, 50 μm. (**e**,**f**) The normalized TH intensity in the GL is significantly lower in the lateral part of the LPS-treated OB than saline-treated OB (**e**), while that of calretinin differs neither in the lateral nor medial part (**f**). Statistical analyses were performed with Mann-Whitney U-test (Mean ± SEM. *p < 0.05, compared to saline-treated control).

To examine the potential effects of persistent rhinitis on OB neurons, we first focused on the periglomerular cells that are interneurons located in the GL and receive synaptic input from OSN axons in the glomerulus. Periglomerular cells can be classified into several subgroups based on molecular expression patterns^29^. Among the molecules expressed by subsets of periglomerular cells, we compared the expression of tyrosine hydroxylase (TH) and calretinin between saline-treated and LPS-treated OBs at Day 21 and found that TH expression in the GL was weaker in the lateral part of the LPS-treated OB than in the saline-treated OB (Fig. 6a–d). The intensity of TH immunofluorescence in the GL (normalized to the non-treated side of the same animal; see Methods) was significantly lower in the lateral part of the LPS-treated OB (67.6 ± 5.9%) compared to the corresponding layer of the saline-treated OB (99.5 ± 5.0%) (Fig. 6e). However, no obvious difference was found in the calretinin expression between LPS-treated and saline-treated OBs (Fig. 6a–d). The normalized calretinin intensity did not significantly differ between LPS-treated and saline-treated OBs in either the lateral or medial part (Fig. 6f).

**Figure 7:**
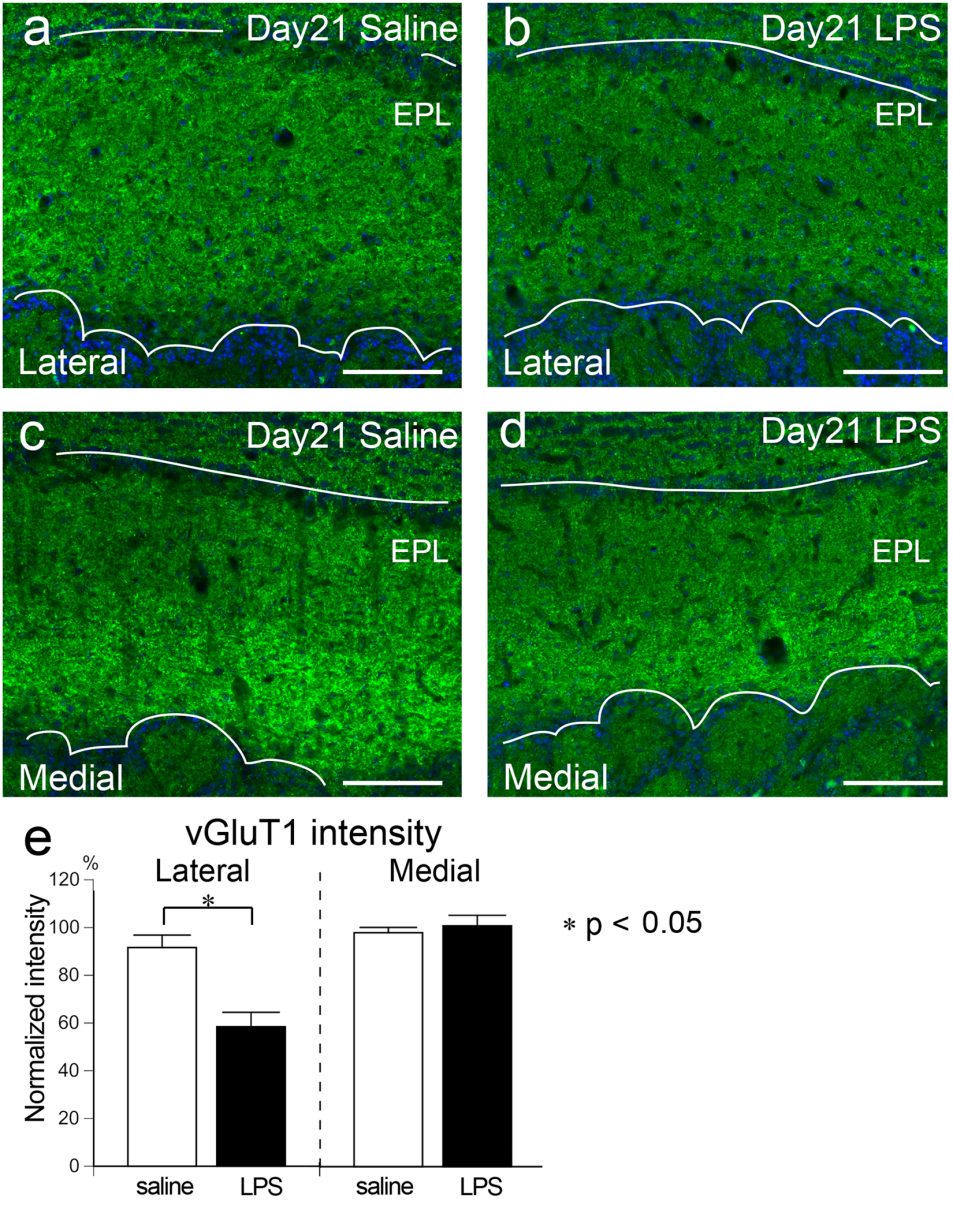
Reduction of vGluT1 signal in the EPL of the LPS-treated OB. (**a**–**d**) Images from the EPL of saline- (**a**,**c**) and LPS-treated (**b**,**d**) OBs stained with vGluT1 (green). VGluT1 signal is weaker in the lateral part of LPS-treated OB (**b**) compared to that of saline-treated OB (**a**) at Day 21, but not in the medial part (**c**,**d**). All nuclei are stained with DAPI (blue). Scale bars, 100 μm. (**e**) The normalized vGluT1 intensity in the EPL is significantly lower in the lateral part of the LPS-treated OB compared to that of the saline-treated OB. No difference is found in the medial part. Statistical analyses were performed with Mann-Whitney U-test (Mean ± SEM. *p < 0.05, compared to saline-treated control).

Mitral and tufted cells are OB projection neurons whose primary dendrites are innervated by OSN axons in the GL. Primary dendrites of mitral/tufted cells make dendrodendritic synapses with periglomerular cells and granule cells in the GL and EPL, respectively. Vesicular glutamate transporter 1 (vGluT1) is a presynaptic protein expressed by mitral/tufted cells and localized to the dendrodendritic synapses; therefore, vGluT1 signals are evident in the GL and EPL of the OB^30^ (Fig. 7a). We found that the vGluT1 immunofluorescence at Day 21 was weaker in the lateral part of the LPS-treated OB, but not in the medial part, compared to the saline-treated OB (Fig. 7a–d). The normalized intensity of vGluT1 in the EPL was significantly lower in the lateral part of the LPS-treated OB (58.4 ± 6.1%) compared to the corresponding layer of the saline-treated OB (91.6 ± 5.2%). The intensity did not differ between LPS-treated and saline-treated OBs in the medial part (100.9 ± 4.4% and 98.0 ± 2.2%, respectively) (Fig. 7e).

**Figure 8:**
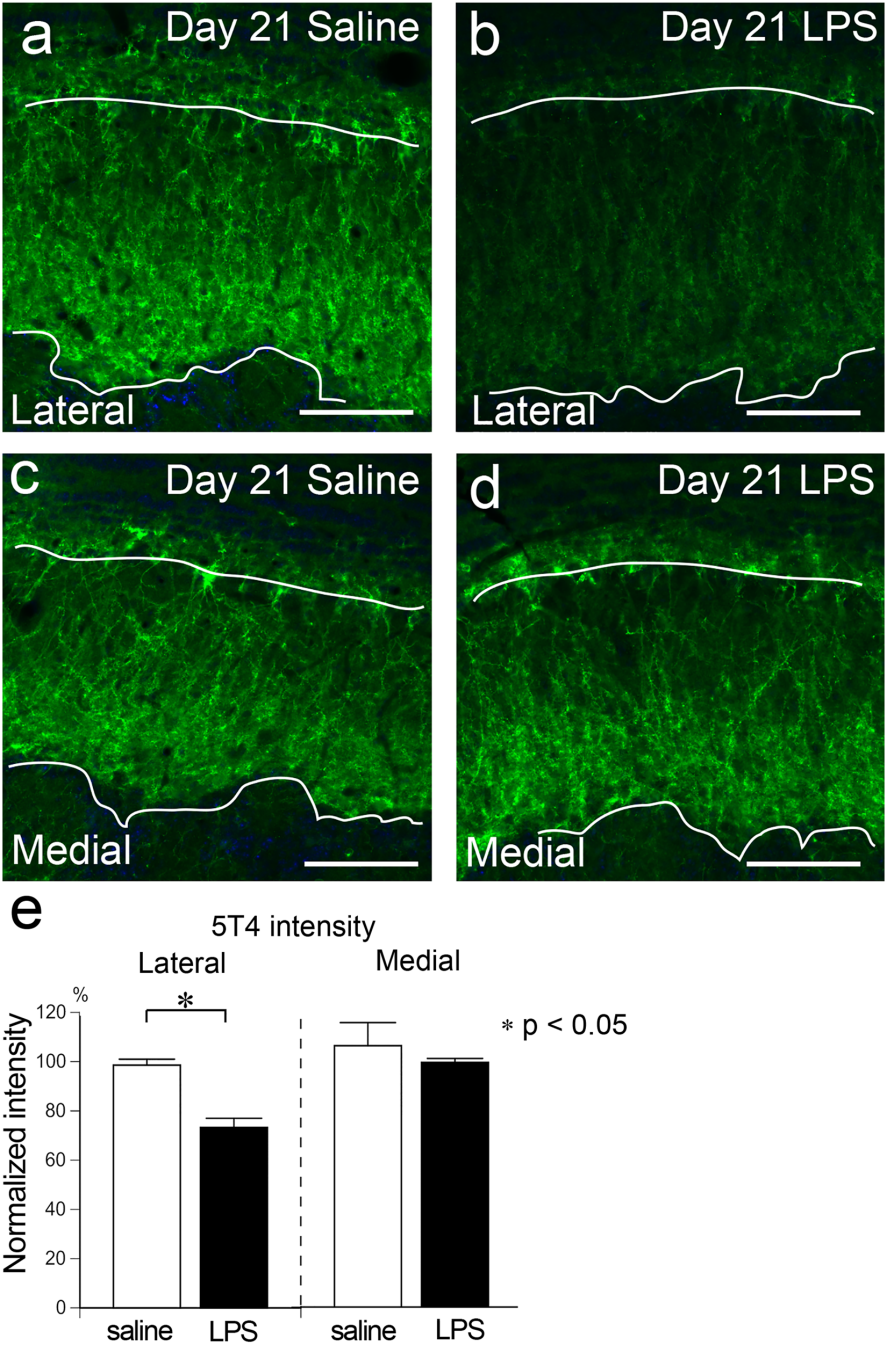
Reduction of 5T4 signal in the EPL of the LPS-treated OB. (**a**–**d**) Images from the EPL of saline- (**a**,**c**) and LPS-treated (**b**,**d**) OBs stained with 5T4 (green). 5T4 signal is weaker in the lateral part of LPS-treated OB (**b**) compared to that of saline-treated OB (**a**) at Day 21, but not in the medial part (**c**,**d**). Scale bars, 100 μm. (**e**) The normalized 5T4 intensity in the EPL is significantly lower in the lateral part of the LPS-treated OB compared to that of the saline-treated OB. No difference is found in the medial part. Statistical analyses were performed with Mann-Whitney U-test (Mean ± SEM. *p < 0.05, compared to saline-treated control).

Finally, we examined the effects of persistent rhinitis on granule cells. 5T4 is a molecule that is expressed in a subset of granule cells that project dendrites to the superficial EPL (Fig. 8a)^31^. Compared to saline-treated OB, we found a pronounced reduction of 5T4 expression in the EPL at Day 21 in the lateral part of the LPS-treated OB, but not in the medial part (Fig. 8a–d). The normalized intensity of 5T4 was significantly lower in the lateral part of the LPS-treated OB (73.3 ± 3.7%), compared to the corresponding layer of the saline-treated OB (98.6 ± 2.4%). There was no difference between the medial part of the LPS-treated and saline-treated OBs (99.4 ± 1.6% and 106.1 ± 9.4%, respectively) (Fig. 8e). These results suggest that dendrodendritic synapses in the OB were lost in response to persistent rhinitis.

## Discussion

In the present study, we investigated the histopathological changes in the OM and OB induced by intranasal LPS-initiated persistent rhinitis. Intranasal LPS administration induced leukocyte infiltration, IL-1β production, and loss of OSNs in the OM. Further, the inflammatory response was transmitted to the OB, where intranasal LPS administration caused microglial activation, astrocytic gliosis, decreased TH expression in periglomerular cells and loss of dendrodendritic synapses formed between mitral/tufted cells and granule cells. We demonstrated the detrimental effects that persistent rhinitis exerts on OB neurons, suggesting that the OM-OB pathway functions as a brain-immune interface, presumably via cell-cell interactions between OSNs and central neuronal dendrites and those between glial cells and dendrites and/or via some humoral factors.

OM inflammation and specific loss of OSNs were also reported with intranasal administration of toxic agents such as Aspergillus fumigatus^32^, roridin A^15^, cigarette smoke^33^, satratoxin G^16^ and polyinosinic-polycytidylic acid (Poly IC)^34^. Infiltration of inflammatory cells and loss of OMP-positive OSNs are also reported in human patients with chronic rhinosinusitis^35^. However, the cellular and molecular mechanisms underlying OSN loss remains unknown. Damage to OSNs caused by intranasal Poly IC administration is blocked by a neutrophil elastase inhibitor and is suppressed in cyclophosphamide-induced neutropenic mice, suggesting that elastase released from neutrophils contributes to the OSN damage^34^. In the present study, LPS administration also induced neutrophil infiltration into the OM. Therefore, neutrophil elastase may also be involved in the LPS-induced OSN loss. Cytokines are other candidates for the OSN loss. CD11b- and Iba-1-positive cells that infiltrated the OM produced IL-1β in response to acute rhinitis. IL-1β is known to trigger apoptosis of target cells that express the IL-1β receptor (IL-1R1). *In vitro* cell apoptosis induced by hypoxia, trophic factor deprivation or TNFα treatment can be blocked by pretreatment with IL-1R1 antagonist^36^. IL-1β, released from LPS/interferon gamma-activated microglia, arrests the cell cycle of neural precursor cells, leading to apoptosis via the tumor suppressor p53-dependent pathway^37^. Our preliminary immunohistological data suggest that IL-1R was expressed by immature OSNs. Therefore, IL-1β released from CD11b- and Iba-1-positive cells infiltrating the OM may be involved in the LPS-induced specific OSN loss. Regeneration of OSNs and recovery of OE structure are other interesting questions awaiting further studies.

The damage caused by intranasal LPS administration was prominent in the lateral OE. Similar geographical patterns of OE damage have been reported in studies with intranasal administration of satratoxin G, roridin A and Poly IC^15, 16, 34^. Given that intranasally administered Trypan Blue was predominantly localized to the lateral part of the nasal cavity (Supplementary Fig. 1), restricted access of the solution to the lateral part may be an underlying mechanism of region specificity of the damage. Further studies about the OE structure, solution removal by mucus flow, and airflows in the nasal cavity would be necessary for understanding the mechanism of restricted solution access. On the other hand, there is a clear boundary between dorsal and ventral zones of rodent OE. For example, several molecules, such as OCAM, NAD(P)H quinone dehydrogenase (NQO1), and olfactory specific medium-chain acyl-CoA synthase (O-MACS), are differentially expressed by OSNs located in these zones^28, 38, 39^, and differences in cellular homeostasis are also suggested^40^. Therefore, it is also possible that differences in molecular and cellular properties between dorsal and ventral OE caused the spatial heterologous effects in response to the LPS exposure.

In the present study, we found that neurodegenerative changes also occurred in the lateral part of the OB. Given that OSNs located in the lateral part of the OE innervate the lateral part of the OB, the degeneration of OSNs is very likely to be causally associated with the neurodegeneration of the OB. In the present study, the TH immunofluorescent intensity was reduced in periglomerular cells following intranasal LPS administration. The TH expression level of periglomerular cells represents neuronal activity in response to olfactory stimulation, and it has been reported that the deprivation of sensory input by naris closure produces a decrease in TH expression and TH activity in periglomerular cells^41^. Thus, our result indicates that periglomerular cell activity was lowered due to the loss of input from OSNs caused by persistent rhinitis.

We suspect that the degeneration of OSNs and the microglial activation with astrocytic gliosis in the ONL and GL of the OB are causally-related, since the ONL and GL are rich in OSN axons. It is known that glial cells are activated in response to axonal degeneration. For example, intraperitoneal or hippocampal injection of kainic acid, a potent neuroexcitatory amino acid, caused pyramidal cell death in the cornu ammonis 3 (CA3) region, resulting in the degeneration of axons in the CA1 region^42^. This axonal degeneration induced activation of microglia and hypertrophic changes of astrocytes in the stratum radiatum in the CA1^42–45^. Activated microglia then released a variety of toxic substances such as prostaglandins, NO, and pro-inflammatory cytokines that may injure nearby cells^46–48^. Microglial activation and astrocytic hypertrophy of the OB shown in the present study were very likely a response to axonal degeneration of OSNs. Activated microglia might release a variety of substances to initiate neuroinflammatory responses in the OB, which lead to degenerative changes in OB neurons.

The present study revealed that the intensities of vGluT1 and 5T4 expression decreased in the lateral part of the EPL of LPS-treated OB. The reduced expression of vGluT1 and 5T4 in the EPL indicated a loss of dendrodendritic synapses formed between mitral/tufted cells and granule cells^30, 31^. These data suggest that the loss or degeneration of OSNs and/or subsequent glial activation in the OB exert detrimental effects on the synapses formed by the second-order neurons and interneurons of the OB in response to a nasal insult.

We also observed Ly-6G-positive and CD11b-positive cells in the GL and EPL of LPS-treated OB at Days1 and 3 (Supplementary Fig. 3), whereas CD3-positive T cells were observed at Day 21. A mild-to-moderate influx of neutrophils into the GL 7 days after satratoxin G instillation has been previously reported^16^. Therefore, rhinitis induces local encephalitis, in which activated leukocytes may affect the brain cells by cell-cell interaction. How the leukocyte infiltration of the OB is involved in persistent rhinitis-induced neurodegenerative changes in the OB or the route of entry for these infiltrates into the OB remains to be determined. There is a possibility that blood-borne leukocytes enter the OB by crossing the blood-brain barrier, which might be structurally compromised by acute inflammation. Another possibility is that once leukocytes enter the OE, they gain access to the OB by using OSN axons as a migratory scaffold^16^.

One of the most striking findings in the present study is the influence of peripheral inflammation on the CNS projection neurons. This is analogous to previously reported findings that severe corneal inflammation induced by chronic instillation of benzalkonium chloride damages primary sensory neurons in the trigeminal ganglion, leading to the activation of second-order neurons, interneurons and glial cells in the brainstem and to the production of pro-inflammatory cytokines^49^. The activation of second-order neurons with brainstem inflammation further activates motor neurons of the facial nerve nucleus. Similarly, in response to peripherally administered LPS or IL-1β, vagal afferents mediate the activation of the brainstem, hypothalamus and limbic structures that represent primary and secondary projection areas of the vagus nerves^50, 51^. When LPS or IL-1β is injected into the soft palate, a febrile response is induced in rats, which is attenuated by the transection of the glossopharyngeal nerves^52^. These previous findings, together with the present study, suggest that peripheral nerves can convey inflammatory information to CNS projection neurons, which may further transmit the inflammatory information to deeper brain regions.

The fact that the synapses on mitral/tufted cell dendrites were lost in our persistent rhinitis model engenders interest into whether and how the projection axons, emanating from mitral/tufted cells, are affected by persistent rhinitis. We hypothesize that long projection axons of mitral/tufted cells undergo degenerative changes and exert negative influences on their innervating brain regions. Mitral/tufted cells send axons to the olfactory cortex including the anterior olfactory nucleus, olfactory tubercle, piriform cortex, entorhinal cortex and amygdala^53–56^. Interestingly, olfactory dysfunction is one of the common and early symptoms of several neurodegenerative diseases including Parkinson’s disease and Alzheimer’s disease^57, 58^, and the olfactory cortex is vulnerable in these diseases^59, 60^. Moreover, exposure to environmental toxins increases the risk for these neurodegenerative diseases, and allergic rhinitis is associated with development of Parkinson’s disease later in life^61^. In summary, the present study is an important first step to determine the effects of olfactory inflammation on the brain, as well as the potential contribution of the olfactory pathway to the pathogenesis of neurodegenerative diseases.

## Methods

### Animals

Male C57BL/6 J mice at 7 weeks of age were obtained from the Jackson Laboratory (Bar Harbor, ME, USA) and acclimated for 1 week in our animal facility. To model rhinitis, mice received a 10 μL administration of physiological saline or LPS from Escherichia coli O55:B5 (Sigma, St. MO, USA) dissolved in physiological saline (1 mg/mL) into a unilateral nostril under a deep anesthesia with isoflurane three times per week (every other day). Mice were sacrificed for histological preparation at 1, 3, 7, 14, or 21 days after the first administration. To determine the distribution of solution in the nasal cavity, mice received a single administration of 0.4% trypan blue (10 μL) in a unilateral nostril under deep anesthesia with isoflurane, and then sacrificed 30–60 min after the administration. All protocols were approved by, and all methods were performed in accordance with the guidelines of, the Institutional Animal Care and Use Committee (IACUC) of Penn State Hershey and Guide of The Association for Assessment and Accreditation of Laboratory Animal Care International (Frederick, MD, USA).

### Histological preparation

Mice were deeply anesthetized with ketamine (100 mg/kg) and xylazine (10 mg/kg) dissolved in physiological saline and perfused transcardially with 0.1 M phosphate buffered saline (PBS) followed by 80 mL of 4% paraformaldehyde (PFA) at a rate of 8 mL/min. Heads were removed and placed in the same fixative at 4 °C overnight. For each head, the caudal half of the calvaria (posterior to the bregma) was removed. The rostral half of the calvaria (anterior to the bregma) and the nasal bone were remained to preserve the nasal mucosa and its continuity with the brain. The rostral brain and nasal cavity were then placed en block in 0.45 M ethylenediaminetetraacetic acid (EDTA) in PBS at 4 °C for 2 days for decalcification, cryoprotected with 30% sucrose (wt/vol) at 4 °C overnight, and embedded in optimal cutting temperature compound (OCT, Sakura Finetek USA, Torrance, CA, USA). Frozen sections were cut coronally using a cryostat (Leica Biosystems Inc., Buffalo Grove, IL, USA) at 20 μm thickness. Sections were dried and kept in a freezer until they were subjected to staining.

### Immunohistological analysis

For immunofluorescence staining, frozen sections were rehydrated in tris-buffered saline with 0.3% Triton-X (TBST, pH 7.4) for 15 minutes. Selected sections to be stained with primary antibodies for CD3, OCAM and 5T4 were pretreated in sodium citrate (pH 6.0) at 65 °C for 60 min and then rinsed with TBST for the sake of antigen retrieval. Sections stained with primary antibody for SOX-2 were pretreated in 0.025 M HCl at 65 °C for 60 min, immersed with 0.1 M borate buffer (pH 8.5) for 10 min and then rinsed with TBST for the sake of antigen retrieval. After immersion in 5% normal donkey serum in TBST (blocking buffer) to block non-specific binding sites, sections were incubated with primary antibodies in blocking buffer at 4 °C overnight. Primary antibodies used in the present study are listed in Supplementary Table 1. Sections were then incubated with Alexa Fluor 555- or 488-conjugated anti-rabbit, anti-rat, anti-mouse or anti-goat IgG secondary antibodies (Thermo Fisher Scientific, Waltham, MA, USA, in accordance with the species origin of primary antibodies) at room temperature for 60 min. Nuclei were counterstained with 4′, 6-diamidino-2-phenylindole (DAPI; Thermo Fisher Scientific). Sections were mounted and coverslipped with Gel/Mount mounting medium (Electron Microscopy Sciences, Hatfield, PA, USA) and observed with a fluorescence microscope (Axio Scope. A1, Carl-Zeiss, Jena, Germany) equipped with a CCD camera (AxioCam, Carl-Zeiss). Optically sectioned images were captured using a fluorescence microscope with structured illumination (BZ-x710, Keyence, Osaka, Japan)^62^.

### Image analysis and morphometry

All samples were randomly numbered so that the analyses were performed by an experimenter who was blind to sample identities. To determine the geographical spread of tissue damage in the OE, coronal sections of the nasal cavity were made between 360 and 720 μm rostral from the anterior tip of the OB (18 sections). These sections share a similar morphological pattern of the turbinate as shown in Fig. 3a and consistently included the most severely damaged regions in the OE. At least three randomly-selected sections were stained with anti-AC III antibody for each mouse, confirming that the staining patterns were nearly identical among all sections. Subsequently, one section per animal was used for the quantification of AC III. Microphotoimages covering the entire nasal cavity were captured as tiling images with a 10x objective lens. The OE was divided into two areas as follows (see Fig. 3a): Area 1 was the medial part of the OE that covered the most ventral point of the septal OE, upward along the septum and then downward to the bottom (most ventral point) of the first turbinate; and Area 2 was the lateral part of the OE that covered the bottom of the first turbinate, upward along the first turbinate to the midpoint of the second turbinate. The ventrolateral part of the OE was excluded from our analyses, since the AC III expression along the OE is known to be discontinuous in this area. The length of the OE segments labeled with immunofluorescence for AC III and that of the entire OE were measured using Image J software, and the percentage of the summed length of AC III-immunopositive OE segments along the entire OE length was calculated. The percentages were compared between saline- and LPS-treated mice at Days 3, 7, 14 and 21 (n = 3 animals per experimental condition for Days 3, 7 and 14, n = 4 for LPS-treated mice at Day 21 and n = 5 for saline-treated mice at Day 21).

The number of mature and immature OSNs were quantified by counting cells immunopositive for OMP and GAP43, respectively, using coronal sections of the nasal cavity. Sections were made between 360 and 720 μm rostral from the anterior tip of the OB (18 sections). At least three randomly-selected sections were stained with anti-OMP and GAP43 antibodies for each mouse, confirming that the staining patterns were nearly identical among all sections. Subsequently, one section per animal was used for the quantification of OMP/GAP43-positive cell. Microscopic photoimages were captured from 2 regions of the OE segments in Area 2 as indicated with a rectangle in Fig. 3a using a 40x objective lens. DAPI-stained nuclei of cells with OMP-immunopositive cytoplasm and those with GAP43-immunopositive cytoplasm were counted separately. Cell counts were divided by the length of the OE to calculate the cell densities (cells/mm). The density of OMP-immunopositive cells and that of GAP43-immunopositive cells were separately analyzed and compared between saline- and LPS-treated mice at Days 3, 7, 14 and 21 (n = 3 animals for all experimental conditions, except n = 4 for OMP-immunopositive cells in LPS-treated mice at Day 21 and n = 5 for GAP43-immunopositive cells in LPS-treated mice at Day 21).

To quantify the immunofluorescent intensity of OCAM, TH, calretinin, vGluT1 and 5T4 in the OB at Day 21, microphotoimages of sections were taken with a 20x objective from the lateral and medial parts of the saline- or LPS-treated OBs. Coronal sections were made between 600 and 900 μm rostral from the anterior tip of the AON (15 sections). These sections shared a similar morphological pattern as shown in Fig. 4a and included the most significant gliosis in the OB. Two sections were stained with anti-OCAM antibodiy and three sections were stained with anti-TH, calretinin, vGluT1 or 5T4 antibodies for each mouse and used for quantitative analyses. Microphotoimages were also taken from the corresponding parts of the contralateral non-treated OBs for the sake of normalization to reduce the contributions of variations among animals and sections. The same exposure time was applied to capture the images from the same animal. Using Photoshop software (Adobe Systems, Sun Jose, CA, USA), the immunofluorescent intensity of each antigen obtained from the ipsilateral OB were normalized by dividing them by the intensity obtained from the contralateral OB on the same section. The mean of the normalized intensity was calculated for each mouse. The values were compared between saline- and LPS-treated mice (n = 4 animals for LPS-treated mice and n = 5 for saline-treated mice at Day 21 for OCAM analysis; n = 4 each for all experimental conditions for TH, calretinin and vGluT1 analyses; and n = 3 each for all experimental conditions for 5T4 analysis).

To measure the geographical area of the OB labeled with immunofluorescence for Iba-1 at Days 3, 7, 14 and 21, coronal sections were made between 600 and 900 μm rostral from the anterior tip of the AON (15 sections). At least three randomly-selected sections were stained with anti-Iba-1 antibody for each mouse, confirming that the staining patterns were nearly identical among these sections. Subsequently, one section was used for the quantification of Iba-1-positive area. Microphotoimages were taken with a 20x objective from the lateral and medial parts of the LPS- or saline-treated OBs. The same exposure time was applied to capture the images from the same animal. Using Photoshop software, immunofluorescence for Iba-1 was converted to binary image and the white pixels were defined as the Iba-1-immunopositive pixels. The percentage of the Iba-1 + area was calculated by dividing the Iba-1-immunopositive pixel count with total pixel count in the entire area of interest (ONL, GL and EPL). The percentages of Iba-1 positive area were compared between saline- and LPS-treated OB in the lateral part at Days 3, 7, 14 and 21 (n = 3 animals per experimental condition).

### Statistics

The percentage of AC III-immunopositive length, OMP-immunopositive cell density, GAP43- immunopositive cell density, percentage of Iba1-immunopositive area, and immunofluorescent intensities of OCAM, TH, calretinin, vGluT1 and 5T4 were analyzed using Mann-Whitney U-test at each day to compare the values between saline- and LPS-treated OE or OB. All statistical analyses were performed using Statistica software (Statistica, Tulsa, OK, USA). All numbers shown in the Results section are expressed as Mean ± SEM.

### Ethics approval and consent to participants

The studies presented in this manuscript were previously approved by the Institutional Anima Care and Use Committee (IACUC) of Penn State Hershey.

## Electronic supplementary material

Supplementary Information
