# Supplementary material for: Lipopolysaccharide-initiated persistent rhinitis causes gliosis and synaptic loss in the olfactory bulb
Source: Sci Rep. 2017 Sep 14;7:11605. doi: 10.1038/s41598-017-10229-w (PMC5599676; doi:10.1038/s41598-017-10229-w)
Supplement: Supplementary file 1 — Supplementary Information [file 41598_2017_10229_MOESM1_ESM.pdf]

Supplementary Data

Lipopolysaccharide-initiated persistent rhinitis causes gliosis and synaptic loss in the olfactory bulb

Sanae Hasegawa-Ishii[1], Atsuyoshi Shimada[2] and Fumiaki Imamura[1]

[1]Pennsylvania State University College of Medicine, 500 University Drive, Hershey, PA 17033, USA

[2]Faculty of Health Sciences, Kyorin University, 5-4-1 Shimorenjaku, Mitaka, Tokyo 181-8612, Japan

Sanae Hasegawa-Ishii; sishii@pennstatehealth.psu.edu

Atsuyoshi Shimada; ats7@ks.kyorin-u.ac.jp

Fumiaki Imamura; fimamura@pennstatehealth.psu.edu

Corresponding author: Fumiaki Imamura (fimamura@pennstatehealth.psu.edu)

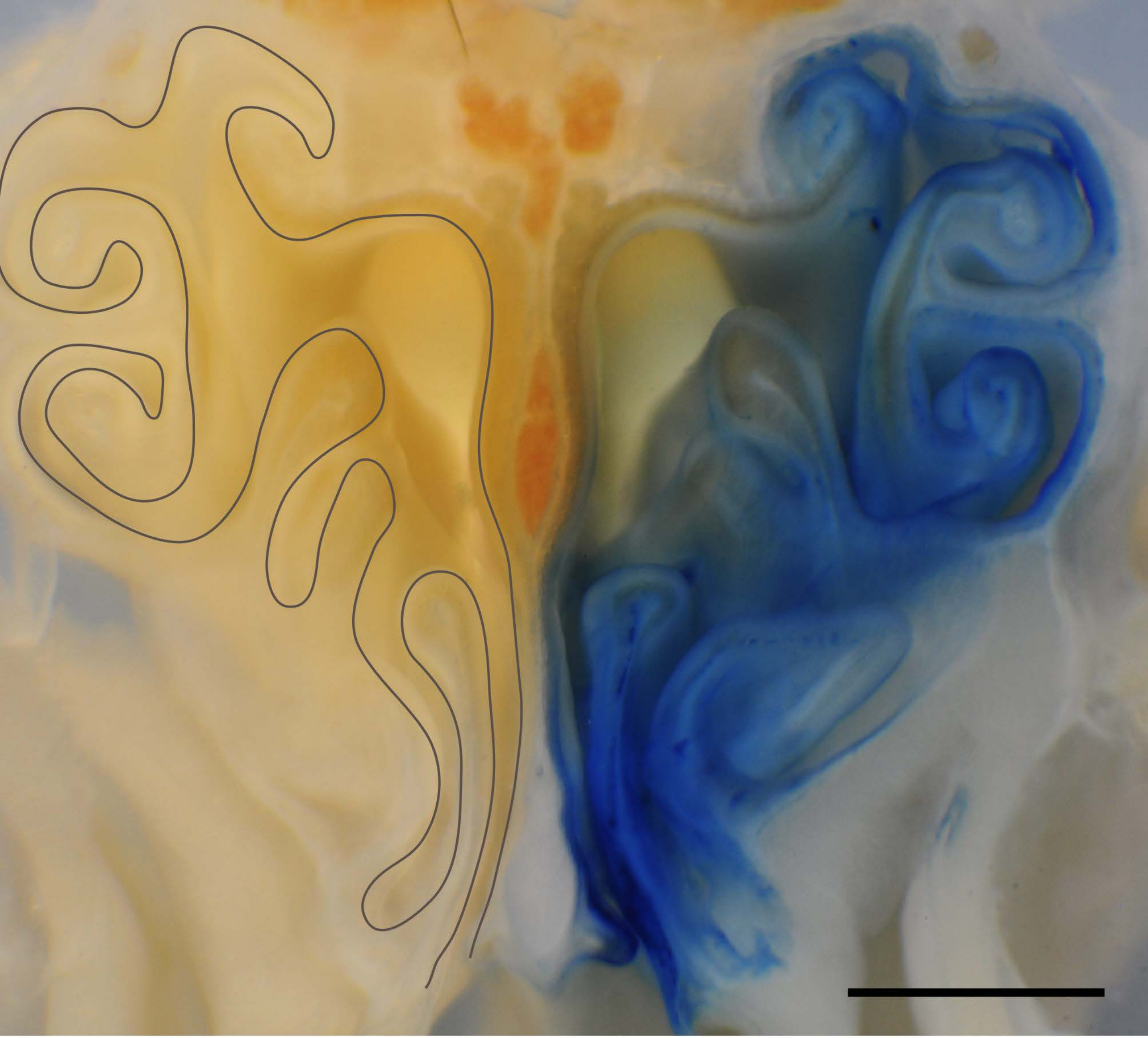

Supplementary Figure1. Intranasal administration of Trypan Blue. Blue stain is localized to the lateral part of the OE, while the medial part is weakly stained. Scale bar, 1 mm.

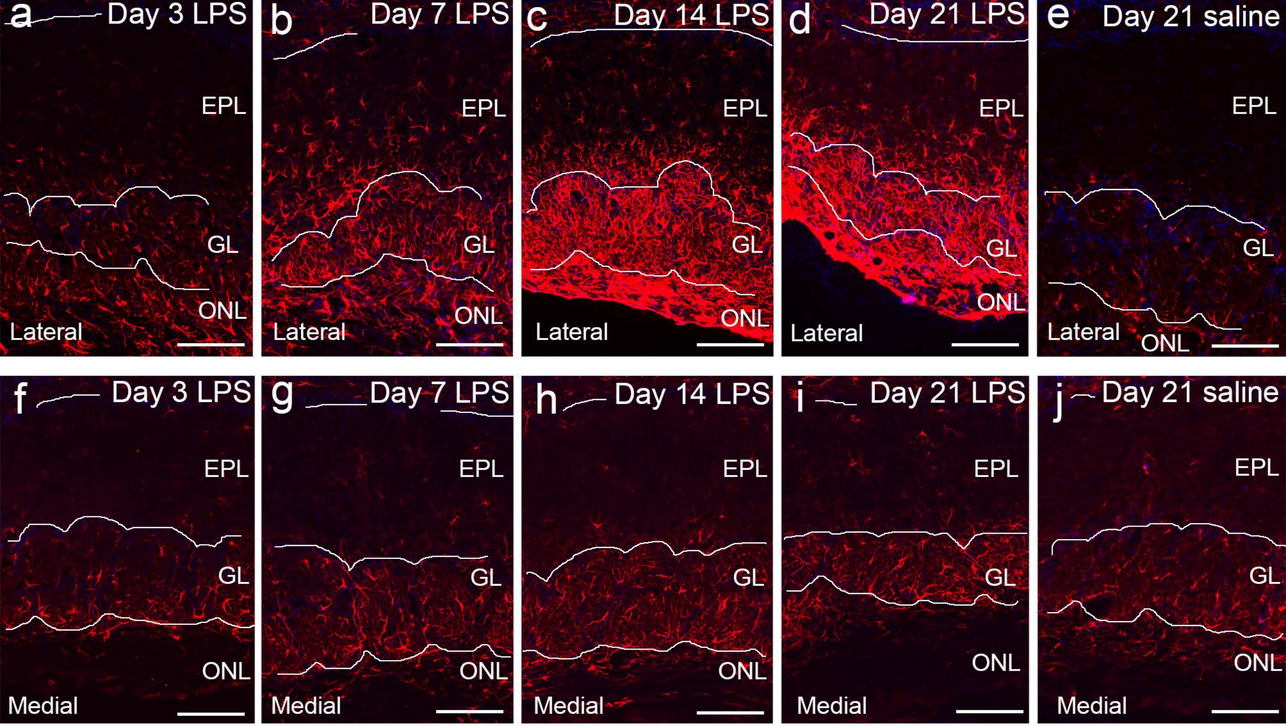

Supplementary Figure 2. Activation of astrocytes in the lateral LPS-treated OB. (a-e) Hypertrophy and accumulation of GFAP immunopositive astrocytes (red) are observed in in the ONL, GL and EPL of the lateral part of the LPS-treated OB (f-j) GFAP immunopositive astrocytes (red) in the medial part of the LPS-treated OB do not change their morphology or distribution. All nuclei are stained with DAPI (blue). Scale bars, 100 μm.

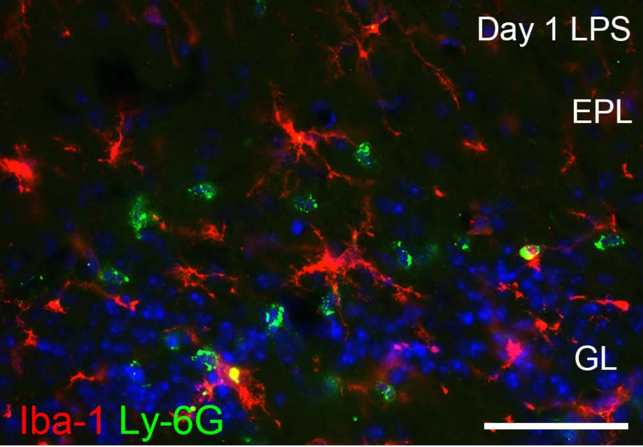

Supplementary Figure 3. Infiltration of inflammatory cells in the OB. Ly-6G-positive cells (green) are found in the GL and EPL of LPS-treated OB at Day 1. Microglia are stained with anti-Iba-1 antibody (red). All nuclei are stained with DAPI (blue). Scale bar, 50 μm.

Supplementary Table 1. List of primary antibodies

Antibody against	Host	Dilution	Source	Immunogen
ACIII	Rabbit	1:200	Santa Cruz Biotechnology (Dallas, TX, USA)	AA 1-270 mapping at the N-terminus of adenylate cyclase III of human origin
Ly-6G	Rat	1:200	AdipoGen (Liestal, Switzerland)	Purified mouse BALB/ c neutrophils, clone Nimp-R14
CD11b	Rat	1:200	Bio Rad Laboratories (Hercules, CA, USA)	Thioglycollate-elicted Peritoneal Macrophages, clone 5C6
CD3	Rabbit	1:100	Thermo Scientific (Fremont, CA, USA)	A synthetic 13-mer peptide corresponding to aa 156-168 of the epsilon chain of human CD3 protein, clone SP7
Iba-1	Rabbit	1:200	Wako Chemicals USA (Richmond, VA, USA)	A synthetic peptide corresponding to the Iba-1 C-terminal sequence, (PTGPPAKKAISELP)
IL-1β	Goat	1:200	R&D Systems (Minneapolis, MN, ISA)	E. coli-derived recombinant mouse IL-1β/IL-1F2 Val118-Ser269
OMP	Goat	1:1000	Wako Chemicals USA	Rodent OMP
GAP43	Rabbit	1:1000	Novus Biologicals (Littleton, CO, USA)	C-terminal peptide of rat and mouse GAP43 with an N-terminal Cys added to allow chemical coupling to KLH carrier protein
OCAM	Rat	1:200	R&D Systems	Mouse myeloma cell line NS0-derived recombinant mouse OCAM Leu20-Gly700
GFAP	Rabbit	1:1000	Agilent Technologies (Santa Clara, CA, USA)	GFAP isolated from cow spinal cord
TH	Rabbit	1:500	Millipore (Temecula, CA, USA)	Denatured tyrosine hydroxylase from rat pheochromocytoma
Calretinin	Mouse	1:400	Millipore	Recombinant rat calretinin, clone 6B8.2
vGLUT1	Rabbit	1:1000	Synaptic Systems (Gottingen, Germany)	Strep-Tag fusion protein of rat vGluT 1 corresponding to aa 456-560
SOX-2	Rabbit	1:300	Abcam (Cambridge, MA, USA)	A synthetic peptide corresponding to Human SOX2
5T4	Rabbit	1:500	Gift from Dr. K. Mori[31]	Peptide corresponding to C-terminal region of mouse 5T4 (RYEINADPRLTNLSSNSDV)

ACIII, adenylate cyclase III; Ly-6G, leukocyte antigen-6G; Iba-1, ionized-calcium binding adaptor molecule-1; IL-1β, interleukin-1 beta; OMP, olfactory marker protein; GAP43, growth associate protein 43; OCAM; olfactory cell adhesion molecule; GFAP, glial fibrillary acidic protein; TH, Tyrosine hydroxylase; vGLUT1, vesicular glutamate transporter 1
